# Supplementary material for: Cabergoline as a preventive migraine treatment: A randomized clinical pilot trial
Source: PLoS One. 2025 Apr 1;20(4):e0320937. doi: 10.1371/journal.pone.0320937 (PMC11960899; doi:10.1371/journal.pone.0320937)
Supplement: S2 File — (DOCX) [file pone.0320937.s002.docx]

**Cabergoline as a Preventive Treatment for Migraine: An Investigator-Initiated, Randomized, Clinical Trial**

**Principal Investigators**

**Sponsor/Investigator**

Professor Jens Otto Lunde Jørgensen, MD

Department of Clinical Medicine, Aarhus University

Department of Endocrinology, Aarhus University Hospital,

Email: joj@clin.au.dk

Phone: +45 20 72 73 83

**Project Coordinator and Investigator**

Dr. Astrid Hjelholt, MD

Department of Clinical Medicine, Aarhus University.

Steno Diabetes Centre, Aarhus, Aarhus University Hospital.

Email: ajh@clin.au.dk

Phone: +45 24 80 06 64

**Collaborators**

Professor Flemming W. Bach, MD

Department of Clinical Medicine, Aarhus University

Professor Troels Staehelin Jensen, MD

Department of Clinical Medicine, Aarhus University

Associate professor Helge Kasch, MD

Neurology Department, Aarhus University Hospital

Professor Henrik Støvring

Steno Diabetes Centre, Aarhus, Aarhus University Hospital.

**Study synopsis**

| **Name of investigational treatment**  Cabergoline 0.5 mg once weekly |
| --- |
| **Title of study**  Cabergoline as a Preventive Treatment for Migraine: An Investigator-Initiated, Randomized, Clinical Trial |
| **Sponsor**  Jens Otto Lunde Jørgensen, Professor, Dr.Med., MD, Department of Clinical Medicine, Aarhus University, Department of Endocrinology, Aarhus University Hospital. |
| **Coordinating Investigator**  Astrid Johannesson Hjelholt, MD, PhD, Steno Diabetes Center Aarhus, Aarhus University Hospital, Denmark, Dept. of Clinical Pharmacology, Aarhus University Hospital, Denmark. |
| **Collaborators**   - Professor Flemming W. Bach, Dr.Med., MD   Department of Clinical Medicine, Aarhus University   - Professor Troels Staehelin Jensen, Dr.Med., MD   Department of Clinical Medicine, Aarhus University   - Associate professor Helge Kasch, Dr.Med., MD   Neurology Department, Aarhus University Hospital   - Professor Henrik Støvring, Dr.Med.   Steno Diabetes Centre, Aarhus, Aarhus University Hospital. |
| **Study center**  Aarhus University Hospital |
| **Objectives**   To compare cabergoline 0.5 mg once weekly with placebo in the preventive treatment of migraine in a single center, prospective, randomized, placebo-controlled clinical trial. |
| **Methodology**  Following informed consent, participants are randomized in a 1:1 fashion to cabergoline 0.5 mg once weekly or placebo for 12 weeks. |
| **Number of subjects**  36 |
| **Inclusion criteria**  Patients with migraine and more than 6 migraine days per month  and  ≥18 years of age.  and  written informed consent. |
| **Exclusion criteria**   - Known or suspected heart valve disease - Severe untreated hypertension - Treatment-requiring psychiatric diagnosis - Treatment with dopamine receptor agonists - Treatment with medications known to interact with Cabergoline (dopamine D2 receptor antagonists, macrolides, and itraconazole) - Pregnant or breastfeeding women - According to Investigator discretion individuals deemed unlikely to comply with prescribed study procedures, restrictions, or prerequisites will be excluded. Additionally, the Investigator possesses the authority to identify and exclude, at their discretion, any conditions that may render a participant's involvement in the study unsafe or inappropriate. |
| **Primary outcome measure**  Change in Monthly Migraine Days (MMD) between the baseline period and the final 28 days of the treatment period. |
| **Secondary outcome measures**   - ≥ 50% reduction in MMD - PGIC (Patients Global Impression of Change) measured on a numerical rating scale 0-10 (Likert scale 0-10; where 0=very much worse, 10=very much better, 5=no change) - use of acute migraine–specific medication (triptans) - change from baseline in MIDAS score and HIT-6 score |
| **Exploratory outcome measure**  Change in serum prolactin level from baseline |
| **Safety**  To ensure participant safety, phone calls are scheduled after four and eight weeks, specifically inquiring about side effects. Throughout the trial and up to four weeks after, participants can contact one of the trial coordinators if they experience any side effects from the trial medication. |

**1. Abbreviations**

CGRP neuropeptide calcitonin gene-related peptide

HIT-6 headache impact test

MIDAS migraine disability assessment score

MMD monthly migraine days

PGIC patient global impression of change

**2. Study rationale**

**2.1 Background**

Headache, including migraine, is a significant health burden both in Denmark and globally[1, 2]. In Denmark, the estimated lifetime prevalence of migraine is up to 25%. The disease often affects individuals in the working age and is more frequent in women [3, 4]. Migraine is characterized by episodic and unilateral headache, often accompanied by nausea, sensitivity to light and sound, and sometimes aura [5]. The headache lasts between 4 and 72 hours, and a large portion of patients experience recurrent attacks with a frequency of more than eight days per month [6].

**2.2. Pathogenesis of migraine**

The etiology of migraines remains incompletely elucidated; however, the trigeminovascular system, comprising trigeminal neurons and their innervated blood vessels, is deemed pivotal due to the activation and sensitization of afferent neurons with cell bodies situated in the trigeminal ganglion [4, 7, 8]. This activation subsequently engages second-order neurons within the brainstem, encompassing the trigeminocervical complex, and third-order neurons within the thalamus. Nociceptive impulses are then projected to the somatosensory cortex and other cortical regions implicated in pain perception. Concurrently, the release of vasoactive peptides and signaling molecules ensues, inducing local inflammation and dilation of intracranial arteries. Notably, neuropeptide calcitonin gene-related peptides (CGRP) play a significant role in this activation [4]. Preclinical data further propose that activation of the trigeminovascular system leads to parasympathetic outflow to intracranial arteries, including the dural arteries, via the superior salivatory nucleus and the sphenopalatine ganglion. This contributes to the release of diverse signaling molecules, culminating in the dilation of intracranial arteries [4].

**2.3 Treatment of migraine**

The pharmacological management of migraine encompasses acute and preventive strategies, often necessitating a combination of both [9]. Triptans, which selectively activate serotonergic 5-HT1B and 5-HT1D receptors, constitute the primary medications for acute treatment [10]. In the realm of preventive treatment, commonly employed agents include beta-blockers, angiotensin II antagonists, and antiepileptics. Notably, novel therapeutic approaches involve the use of human antibodies targeting calcitonin gene-related peptide (CGRP) and the CGRP receptor, demonstrating efficacy in both acute and preventive settings [11]. Treatment with CGRP antibodies, however, carries a substantial economic burden, and consequently, their utilization in Denmark is limited to prophylactic treatment for chronic migraine in adults, specifically following inadequate response to previous preventive therapies.

**2.4 Cabergoline**

Cabergoline is a dopamine receptor agonist with prolactin-inhibiting effects. Cabergoline has been used for decades and is approved for the treatment of hyperprolactinemic conditions, including prolactin-producing pituitary tumors, inhibition of lactation, gynecomastia, and acromegaly. abergoline is also indicated for the treatment of Parkinson's disease. In addition to its role as a dopamine receptor agonist, cabergoline also exhibits agonistic effects on several serotonin receptors, including 5-HT1B and 5-HT1D [12].

**2.5 Migraine and cabergoline**

In an observational study of patients with hyperprolactinemia and concurrent headaches, cabergoline demonstrated a significant and notable positive impact on headache symptoms [13].

The involvement of dopamine in the pathophysiology of migraine is a subject of ongoing debate [14, 15]. On one hand, dopamine receptor antagonists, such as metoclopramide and domperidone, find application as antiemetics in the acute treatment of migraine, and certain prodromal symptoms are believed to be dopamine-mediated [15]. On the other hand, clinical evidence suggests the efficacy of dopamine receptor agonists, such as bromocriptine, in migraine treatment [16-18]. This aligns with findings indicating that dopamine inhibits nociceptive signals within the trigeminocervical complex in rodent models [19, 20].

Furthermore, dopamine exerts inhibitory effects on the secretion of the pituitary hormone prolactin, and cabergoline and other dopamine receptor agonists, such as bromocriptine, are indicated for treatment of hyperprolactinemia. Prolactin is suggested to be associated with migraines. Rodent experiments propose that prolactin enhances excitability in neurons originating from the trigeminal ganglion, potentially via the CGRP system [21, 22]. Elevated prolactin levels have been observed in female migraine patients [23]. Additionally, hyperprolactinemia, commonly linked to a prolactin-producing pituitary adenoma, frequently coexists with headaches, including migraines [24].

**2.6 Purpose**

In a randomized, placebo-controlled pilot study, we aimed to investigate the effects of cabergoline treatment in patients with migraines experiencing more than six headache days per month compared to placebo.

**3. Methods**

**3.1.1 Design**

This trial is a prospective, randomized, double-blind, placebo-controlled pilot study testing the hypothesis that in patients with migraine, cabergoline 0,5 mg weekly will reduce number of monthly migraine days (MMD) more effectively than placebo. Participants will be randomized to treatment with cabergoline 0.5 mg once weekly or placebo in addition to any existing migraine treatment and followed up after three months (12 weeks) of intervention.

**3.1.2 Randomization**

Thirty-six patients will be randomized to receive treatment with 0.5 mg Cabergoline or placebo once a week. The randomization is conducted in a 1:1 ratio in 2 blocks of 18 patients and performed by the Aarhus University Hospital Pharmacy. The corresponding order of numbering and treatment assignment is specified in a randomization list, which is kept at the Service Production Hospital Pharmacy throughout the entire process. The Hospital Pharmacy also labels both the investigational drug and the placebo. Along with the investigational drug, the Hospital Pharmacy provides sealed code envelopes (corresponding to the randomization) that can be broken for individual participants if it is necessary for the investigator to know the assigned treatment.

**3.1.3 Study program**

The study consists of a 4-week baseline period during which participants will fill out a daily electronic headache diary, first visit (day 1) comprising 2 x questionnaires and blood samples, 12 weeks treatment period during which participants will fill out a daily electronic headache diary, followed by the last visit comprising 2 x questionnaires, PGIC and blood samples. To ensure participant safety and promote compliance, participants will be contacted by phone after four and eight weeks.

**3.1.4 Study medication**

Cabergoline: Cabergoline "Teva," tablets 0.5 mg. Should not be stored at temperatures exceeding 30 °C. Placebo: Placebo tablets, 6 mm. No specific storage conditions.

If a participant forgets to take the investigational medication, the tablet is taken as soon as possible, up to three days after the scheduled time. Any excess medication is subsequently returned to the study coordinator for counting. Unused/expired investigational medication is destroyed.

**3.1.5 Blood sample**

Blood samples are taken on day 1 and after 12 weeks. A maximum of 30 ml of blood is taken at a time. Bruising may occur after blood sampling. Infection is extremely rare.

**3.2 Patients**

A total of 36 patients with more than six migraine days per month and with stable migraine treatment will be included in the study.

**3.2.1 Patient recruitment and inclusion**

Participants will be recruitment from the Department of Neurology, Aarhus University Hospital. Additionally, recruitment happen via the official Facebook page of Aarhus University Hospital. Potential participants receive participant information and will subsequently be contacted by the coordinating investigator by phone or email, with their permission.

- - 1. **Inclusion criteria**
- Patients with migraine and more than 6 migraine days per month
- Male or female subjects ≥18 years of age
- Written informed consent.

**3.2.3 Exclusion criteria**

- Known or suspected heart valve disease
- Severe untreated hypertension
- Treatment-requiring psychiatric diagnosis
- Treatment with dopamine receptor agonists
- Treatment with medications known to interact with cabergoline (dopamine D2 receptor antagonists, macrolides, and itraconazole)
- Pregnant or breastfeeding women.
- According to Investigator discretion individuals deemed unlikely to comply with prescribed study procedures, restrictions, or prerequisites will be excluded. Additionally, the Investigator possesses the authority to identify and exclude, at their discretion, any conditions that may render a participant's involvement in the study unsafe or inappropriate.

**3.2.4. Dropout**

Participants can withdraw from the trial at any time without consequences for their future treatment. Data obtained from a withdrawn participant will be utilized, if possible – it depends on when in the study a given participant withdraws. Patients will be withdrawn from the study if:

- The participant wishes to withdraw.
- The investigator determines that the participant cannot adhere to the trial procedures.
- The participant develops severe or intolerable side effects. In such cases, the trial will be stopped for the individual participant, and the investigator will arrange relevant follow-up, including referral for appropriate treatment.
- Conditions arise as described under 'Exclusion Criteria'.
- Changes in the established migraine treatment.
- Pregnancy.

**3.3 Endpoints**

**3.3.1 Primary endpoint**

The primary endpoint was change in MMD between the baseline period and the final 28 days of the treatment period. A migraine day is defined as any calendar day on which the participant has onset, continuation, or recurrence of migraine as recorded in the electronic diary. MMD was defined as number of migraine days per 28 days.

**3.3.2 Secondary endpoints**

- ≥ 50% reduction in MMD
- Patient global impression of change (PGIC) measured on a numerical rating scale 0-10 (Likert scale 0-10; where 0=very much worse, 10=very much better, 5=no change)
- use of acute migraine–specific medication (triptans)
- change from baseline in MIDAS score and HIT-6 score

**3.3.3 Exploratory outcome measure**

Change from baseline in serum prolactin level.

**3.4 Safety**

Participation in this study is considered to be safe. However, there are certain side effects of Cabergoline that need to be taken into account. The most common and severe side effects are mentioned below. To ensure participant safety, phone calls are scheduled after four and eight weeks, specifically inquiring about side effects. Throughout the trial, participants can contact one of the trial coordinators if they experience any side effects from the trial medication. Participants can continue to contact the trial coordinators with concerns about side effects up to four weeks after the final visit and the end of treatment (the substance is considered to be out of the body after 14 days; 5 x the half-life of Cabergoline).

**3.4.1 Cabergoline**

I this trial, participants randomized to cabergoline treatment will recieve 0.5 mg/week, totaling 6 mg throughout the trial period. Cabergoline side effects are dose-dependent. According to the product summary, the following side effects have been reported:

Very common (> 10%): Nausea, dyspepsia, gastritis, abdominal pain, fatigue, headache, dizziness.

Common (1 - 10%): Constipation, vomiting, depression, asymptomatic hypotension.

In extremely rare cases, fibrotic diseases, including pericardial and pulmonary fibrosis, have been reported, and a possible association between Cabergoline treatment and fibrotic heart valve disease has been suggested. The association has been observed in Parkinson's patients of advanced age and comorbidity after prolonged high-dose treatment (3 mg/day for more than 6 months ≈ 540 mg). The accumulated dose in this project is 6 mg. Recently, the British Society of Echocardiography, the British Heart Valve Society, and the Society for Endocrinology, based on a review of existing literature, concluded that there is extremely limited evidence that treatment with dopamine receptor agonists at doses used in the treatment of hyperprolactinemia can cause heart valve disease [25].

**3.5 Pregnancy**

Taking Cabergoline during pregnancy or while breastfeeding is discouraged. Pregnancy should be avoided for at least 1 month after treatment is completed due to the drug's long half-life and limited available data on fetal exposure. During the first visit, fertile female participants must undergo a pregnancy test and during the trial and one month afterward, they must use reliable contraception (intrauterine device or hormonal contraception; birth control pills, implants, transdermal patches, vaginal rings, or depot injections).

In this context, a woman is considered fertile from menarche to menopause, unless she is permanently sterilized. Permanent sterilization methods include hysterectomy, bilateral salpingotomy, and bilateral oophorectomy [26].

**4. Statistics and data management**

The data will be collected at Steno Diabetes Center, Aarhus University Hospital, Denmark where data management work and statistical analyses will be performed.

**4.1 Statistical analysis**

Differences between study groups will be assessed with unpaired t-tests or using non-parametric statistics as appropriate. Ordinal variables will be assessed with chi-2 test for trend or Mann-Whitney U test and Pearson’s chi-square test or Fisher's exact test will be used to test differences between proportions. Two-sided statistical significance levels of 5% will be used and estimates will be presented with 95% confidence intervals.

**4.2 Analysis population**

The results will be analyzed according to a modified intention-to-treat principle comprising all patients who undergo randomization and receive the study treatment. Patients who withdraw fter receiving the first study treatment will be censored at the date of withdrawal of consent. Patients who are lost to follow-up will be censored on the day of last study visit. Protocol violations will be monitored continuously. Data collected during the study will be coded so that no subjects can be identified.

**4.3 Sample size calculation**

We have not identified any previous randomized studies investigating the use of cabergoline in patients with migraine. Here we conducted a pilot study. The sample size should be large enough to provide meaningful feedback and detect potential problems, and reflect the diversity and characteristics of the target population. A common rule of thumb is to use a sample size of at least 30 participants. Due to the short duration of the study, we expect a maximum dropout rate of 20%. We will therefore include 18 patients in each intervention group.

# **References**

1. *Global, regional, and national burden of neurological disorders, 1990-2016: a systematic analysis for the Global Burden of Disease Study 2016.* Lancet Neurol, 2019. **18**(5): p. 459-480.

2. Steiner, T.J. and L.J. Stovner, *Global epidemiology of migraine and its implications for public health and health policy.* Nat Rev Neurol, 2023. **19**(2): p. 109-117.

3. Le, H., et al., *Increase in self-reported migraine prevalence in the Danish adult population: a prospective longitudinal population-based study.* BMJ Open, 2012. **2**(4).

4. Ashina, M., *Migraine.* N Engl J Med, 2020. **383**(19): p. 1866-1876.

5. *Headache Classification Committee of the International Headache Society (IHS) The International Classification of Headache Disorders, 3rd edition.* Cephalalgia, 2018. **38**(1): p. 1-211.

6. Chalmer, M.A., et al., *Proposed new diagnostic criteria for chronic migraine.* Cephalalgia, 2020. **40**(4): p. 399-406.

7. Goadsby, P.J., et al., *Pathophysiology of Migraine: A Disorder of Sensory Processing.* Physiol Rev, 2017. **97**(2): p. 553-622.

8. Ashina, M., et al., *Migraine and the trigeminovascular system-40 years and counting.* Lancet Neurol, 2019. **18**(8): p. 795-804.

9. Parikh, S.K. and S.D. Silberstein, *Preventive Treatment for Episodic Migraine.* Neurol Clin, 2019. **37**(4): p. 753-770.

10. Ong, J.J.Y. and M. De Felice, *Migraine Treatment: Current Acute Medications and Their Potential Mechanisms of Action.* Neurotherapeutics, 2018. **15**(2): p. 274-290.

11. de Vries, T., C.M. Villalón, and A. MaassenVanDenBrink, *Pharmacological treatment of migraine: CGRP and 5-HT beyond the triptans.* Pharmacol Ther, 2020. **211**: p. 107528.

12. Kvernmo, T., S. Härtter, and E. Burger, *A review of the receptor-binding and pharmacokinetic properties of dopamine agonists.* Clin Ther, 2006. **28**(8): p. 1065-1078.

13. Kallestrup, M.M., et al., *Prolactinoma-associated headache and dopamine agonist treatment.* Cephalalgia, 2014. **34**(7): p. 493-502.

14. Akerman, S. and P.J. Goadsby, *Dopamine and Migraine: Biology and Clinical Implications.* Cephalalgia, 2007. **27**: p. 1308 - 1314.

15. Charbit, A.R., S. Akerman, and P.J. Goadsby, *Dopamine: what's new in migraine?* Curr Opin Neurol, 2010. **23**(3): p. 275-81.

16. Herzog, A.G., *Continuous bromocriptine therapy in menstrual migraine.* Neurology, 1997. **48**(1): p. 101-2.

17. Mascia, A., J. Afra, and J. Schoenen, *Dopamine and migraine: a review of pharmacological, biochemical, neurophysiological, and therapeutic data.* Cephalalgia, 1998. **18**(4): p. 174-82.

18. Barbanti, P., et al., *Dopaminergic symptoms in migraine: A cross-sectional study on 1148 consecutive headache center-based patients.* Cephalalgia, 2020. **40**(11): p. 1168-1176.

19. Charbit, A.R., et al., *Neurons of the dopaminergic/calcitonin gene-related peptide A11 cell group modulate neuronal firing in the trigeminocervical complex: an electrophysiological and immunohistochemical study.* J Neurosci, 2009. **29**(40): p. 12532-41.

20. Charbit, A.R., S. Akerman, and P.J. Goadsby, *Trigeminocervical complex responses after lesioning dopaminergic A11 nucleus are modified by dopamine and serotonin mechanisms.* Pain, 2011. **152**(10): p. 2365-76.

21. Chen, Y., et al., *An Emerging Role for Prolactin in Female-Selective Pain.* Trends Neurosci, 2020. **43**(8): p. 635-648.

22. Avona, A., et al., *Meningeal CGRP-Prolactin Interaction Evokes Female-Specific Migraine Behavior.* Ann Neurol, 2021. **89**(6): p. 1129-1144.

23. Cavestro, C., et al., *High prolactin levels as a worsening factor for migraine.* J Headache Pain, 2006. **7**(2): p. 83-9.

24. Kreitschmann-Andermahr, I., et al., *Headache and pituitary disease: a systematic review.* Clin Endocrinol (Oxf), 2013. **79**(6): p. 760-9.

25. Steeds, R., et al., *Echocardiography and monitoring patients receiving dopamine agonist therapy for hyperprolactinaemia: A joint position statement of the British Society of Echocardiography, the British Heart Valve Society and the Society for Endocrinology.* Clin Endocrinol (Oxf), 2019. **90**(5): p. 662-669.

26. (CTFG), C.T.F.G., *Recommendations related to contraception and pregnancy testing in clinical trials* 2014.
