# Supplementary material for: Cabergoline as a preventive migraine treatment: A randomized clinical pilot trial
Source: PLoS One. 2025 Apr 1;20(4):e0320937. doi: 10.1371/journal.pone.0320937 (PMC11960899; doi:10.1371/journal.pone.0320937)
Supplement: Attachment — Submitted filename: Response to Reviewers.docx [file pone.0320937.s005.docx]

**Reviewer #1:** The sample size is extremely small and so the study is not qualified as a randomized trial. The results are hardly generalizable. Also results are dominated for female subjects.
Yet from Table 3 we can see a large number of adverse events in the Cabergoline group compared to control group, even for this small study. The drug may not be safely applied.
The results are full of uncertainty. For example, the box plots in Figure 2 are very wide. The chronic migraine subjects even show smaller changes for placebo group than cabergoline group.

We thank the reviewer for taking time to review our manuscript. We agree that the sample size is small as also stated in the discussion as a limitation. However, we disagree that it is not qualified as an RCT since it is randomized, double blind, placebo-controlled, approved by the Ethics Committee system and the National Medicines Agency and conducted in compliance with GCP. Indeed, the majority of the participants were females as are most migraine patients (3:1). We agree that the predominance of female participants limits the generalizability of the results with respect to male patients. **This is a limitation that we have now addressed more explicitly in the discussion section (line 357-359):**

***Finally, the predominance of female participants limits the generalizability of the results, and our findings should therefore be interpreted with caution when applied to male patients.***

Adverse effects are known for cabergoline, but we argue that **the compound is overall very safe, and this particular (very low) dose has been used for the treatment of hyperprolactinemia for > 30 years.** We agree that this is a pilot study and that the placebo effect is sizable, which applies to all migraine studies as also mentioned in our paper. The combination of potentially promising results from this pilot study, the need for additional and affordable migraine treatments, and the safety of cabergoline justifies a new and larger RCT.

**Reviewer #2:** This is a double-blind, placebo-controlled study that investigated the effect of cabergoline 0.5mg for migraine prevention. There are several key issues with this trial that need to be explained.
1. According to the clinicaltrial.gov online registry, this is supposed to be a study of chronic migraine, which is defined as having more of than 15 headache days per month, of which at least 8 are migrainous headaches. Therefore, the inclusion criteria clearly contradict the topic of this registration, and the current study includes both episodic and chronic migraine.

We appreciate your careful review and the opportunity to clarify this important point regarding our study's registration and inclusion criteria.

The clinicaltrials.gov registry entry for this study originally targeted chronic migraine patients. However, following protocol refinement prior to study initiation, we expanded the inclusion criteria to encompass both episodic and chronic migraine patients. This is reflected in the inclusion criteria of the final version of the protocol approved by the authorities and audited by the GCP unit:

- Migraine and more than 6 days with headache every months

The decision to include both episodic and chronic migraine patients was guided by several considerations. First, cabergoline’s potential efficacy spans across the migraine spectrum, and including a broader patient population allows for a more comprehensive evaluation of its effects. Second, as an investigator-initiated trial, this study was exploratory in nature and aimed to provide preliminary data on the safety and efficacy of cabergoline, thereby laying the groundwork for future larger-scale and more targeted investigations.

To address the heterogeneity in the study population, we conducted subgroup analyses to separately evaluate the effects of cabergoline on episodic and chronic migraine patients.

2. There is insufficient evidence to justify the use of cabergoline as a migraine preventive drug, given the potential side effects of gambling/sex/other urges, and others. The authors did not provide sufficient argument for its use in migraine other than case series of comorbid migraine and hyperprolactinemia. The potential binding affinity of cabergoline to 5-HT1B/1D receptors is not a valid argument, as studies with triptans (5-HT1B/1D agonists) do not provide evidence of prevention but may increase the risk of medication overuse headache.

Thank you for raising these points. It is correct that cabergoline may carry side effects as mentioned. Ludomania and hypersexuality AE are, however, exceedingly rare and the cabergoline dose used is very low and similar to that, which has been used to treat hyperprolactinemia for > 30 years. As regards the rationale, we mention a previous (and promising) study using bromocriptine, which also is a dopamine agonist used to treat hyperprolactinemia. Moreover, elevated prolactin levels have been implicated in migraine pathogenesis.

3. The study cohort is small (18 cabergoline vs. 18 placebo). There is no information on the power calculation and whether the study is sufficiently powered to detect a difference between the two cohorts.

This study was designed as a pilot trial. The sample size (18 participants per arm) reflects the exploratory nature of this investigator-initiated trial. While this cohort size limits the ability to detect subtle differences between the groups, it provides valuable preliminary data to inform the design of larger, adequately powered trials. Indeed, pilot studies in this field are recommended in guidelines from the International Headache Society in order to ‘…provide insights that improve the design of fully-powered studies, including a basis for sample size calculations’ (DOI: 10.1177/0333102420941839).

The small sample size is a limitation, which we also state in the discussion. **We have tried to emphasize this further in the revised discussion (line 351-359):**

***The major strength of our study lies in its randomized, placebo-controlled design, which minimizes bias and enhances validity. However, the small sample size remains a limitation, increasing the risk of random variability and the potential of overestimation or underestimation of effects. ... A larger study is needed to substantiate our findings.***

A formal power calculation was not conducted prior to the trial due to the limited existing data on the efficacy of cabergoline for migraine prevention. However, we believe that this pilot study demonstrates the potential of cabergoline in this patient population and provides an important foundation for designing future adequately powered trials.

4. The primary outcome registered on the clinicaltrial.gov was the reduction in migraine days from baseline for the entire study cohort. The result was negative, showing no difference between the two cohorts. The positive result presented in the abstract, is based on the subgroup analysis targeting the episodic migraine subgroup (n=20, 11 for cabergoline vs 9 for placebo), and the result is actually borderline (p=0.04). If the main analysis remains insignificant, the subgroup analysis must be well-founded (better defined a priori) and correct for multiple comparisons, otherwise it is more like data torturing. More importantly, the selective presentation of the subgroup analysis in the abstract is misleading.

Thank you for your feedback regarding the presentation of our results and the subgroup analysis. We appreciate the opportunity to address these concerns.

We fully agree with the importance of avoiding data misrepresentation or "data torturing”, but we disagree that corrections for multiple testing are necessary in this context. The study included only two subgroups (chronic and episodic migraine), and the subgroup analysis is clinically and scientifically sound. As a pilot study, the primary goal was to generate hypotheses and provide preliminary insights as also recommended by the International Headache Society Guidelines (DOI: 10.1177/0333102420941839), and our analysis was consistent with this approach. While the subgroup analysis of chronic versus episodic migraine was not predefined in the protocol, it was guided by the clinical relevance of these subgroups. Chronic and episodic migraines may differ in terms of pathophysiology, disease burden, and potential treatment responses, making this analysis important for understanding the differential effects of cabergoline. We recognize that this limitation should have been stated more clearly in the manuscript, and **we have revised the discussion to reflect this point (line 355-357):**

***… the subgroup analysis in patients with chronic and episodic migraine was not predefined, however it was justified by its clinical relevance.***

We agree that the study did not meet its primary endpoint, a reduction in migraine days from baseline for the entire study cohort. This is clearly stated at the top of the discussion; however, we acknowledge that this was missing in the abstract. **We have revised the abstract to clearly include the results of the primary outcome, noting that no significant difference was observed in the overall cohort (31-35):**

***No significant overall difference in the reduction of monthly migraine days was observed. However, among participants with episodic migraine (n= 20), the mean (SE) reduction in monthly migraine days from baseline to the last 28 days of the treatment period was -5.4 (1.3) with cabergoline compared to -1.8 (0.9) with placebo (p=0.04) [odds ratio: 0.79 (95% CI 0.65 – 0.95), p=0.014].***

**Reviewer #3:** This paper clarified that cabergoline significantly reduced monthly migraine days in episodic migraine without serious adverse effects, supporting further investigation into the use of cabergoline for migraine prevention.

This reviewer finds the paper is well written, and appropriate for publication after minor correction shown as follows.

We thank the reviewer for the positive evaluation of our paper including the support to further investigation into the use of cabergoline migraine prevention.

Line 87: [13th of June] -> [13th of June 2023]

Line 168: “Forty-five” will be replaced with “Fifty-four”

Thank you for the thorough proofreading and for identifying these typos. **We have revised accordingly.**

**Reviewer #4:** Thank you for inviting me to review this manuscript. I believe the authors have conducted a clinical study that is beneficial for migraine patients. Its quality is sufficient to be published after revisions. Below are my suggestions:

We thank the reviewer for the positive evaluation and not least – for pointing out that it may eventually benefit migraine patients.

1. Further Discussion on Study Limitations
Although the discussion section mentions the small sample size as a limitation, other potential influencing factors (e.g., short follow-up duration, sample selection bias) should be explored further. Suggestions for addressing these limitations, such as conducting multicenter or larger-scale studies in the future, can also be included.

We acknowledge that a longer follow-up period might have provided additional insights into the long-term efficacy, safety, and durability of cabergoline's effects. While a study period of 12 weeks is commonly applied in migraine research, **in the revised manuscript, we have emphasized the potential value of extended follow-up periods in future studies (line 359-361):**

***A larger study is needed to substantiate our findings. In this context, a longer follow-up period may be considered to evaluate long-term efficacy, safety, and durability of cabergoline's effects.***

2. Clarify Titles and Annotations in Figures
The data presented in the figures are central to the manuscript, particularly Figures 2 and 3. Strengthening the descriptions in the figure titles and annotations can facilitate readers' quick understanding while highlighting the key conclusions of the study.

Thank you for pointing out the need to clarify the titles and annotations in the Figures. **We have edited the legends as suggested in order to improve the readability.**

3. Enhance the Rigor of Background Argumentation
The background section mentions that "the relationship between dopamine and migraines remains controversial," but the cited references are not sufficiently comprehensive. Recent findings on the role of dopamine in migraine mechanisms should be incorporated to improve the relevance and credibility of the references.

We appreciate the reviewer’s suggestion to enhance the rigor of the background section. **We have revised the text to incorporate recent findings on the role of dopamine in migraine mechanisms (line 62-72):**

***Growing evidence underscores the importance of dopamine in the pathophysiology of migraine, particularly the dopamine D2 receptor, though its exact role remains ambiguous. Dopamine antagonistic drugs are commonly used for acute migraine treatment and certain premonitory symptoms are considered dopamine-driven [1]. On the other hand, the dopamine D2 receptor agonist bromocriptine, an ergot alkaloid derivative, and other dopamine receptor agonists have demonstrated beneficial effects in migraine management [2, 3]. In vivo PET imaging studies have shown an imbalance in dopamine D2/D3 receptor activity during migraine attacks, indicating fluctuations in endogenous dopamine release that correlate with the chronicity and frequency of migraine attacks [4]. Additionally, studies in rodents suggest that D2 receptor activation may influence central nociceptive sensitization in chronic migraine [5, 6].***

4. Improve Readability of Statistical Method Descriptions
The statistical analysis mentions the use of linear regression models and non-parametric tests, but the rationale for selecting these methods is not well explained. Additional clarification on why these methods were chosen and how potential confounding factors were controlled would enhance the methodological clarity.

We appreciate the feedback. In response to the reviewer’s concern, we have simplified the statistical analysis to enhance clarity. We now report means and standard deviations (SDs) for normally distributed variables, and medians with interquartile ranges (IQRs) for non-normally distributed variables. Comparisons between the groups are made using unpaired t-tests for normally distributed variables, and Mann-Whitney U tests for non-normally distributed variables. We have revised the manuscript to reflect this simplified approach and ensure better readability and transparency **(line 158-163).**

***Primary and secondary outcomes were analysed using unpaired t-test and presented as mean ± standard errors (SE), if data were normally distributed. For non-normally distributed data, the Mann Whitney U test was used, and results are reported as medians with interquartile ranges (IQR). Baseline values are presented descriptively as means and standard deviations (SD) for normally distributed variables, or medians with IQR for non-normally distributed variables.***

5. Supplement Detailed Analysis of Adverse Events
While the types and frequencies of adverse events are listed, their impact on patients has not been detailed. Adding analyses based on scales or patient feedback to quantify the impact of adverse events on quality of life could strengthen this aspect of the manuscript.

In our study, adverse events were recorded by type and frequency, however, we recognize the value of patient feedback to quantify the impact of adverse events on quality of life. While specific quality-of-life measures related to adverse events were not collected, the Patients’ Global Impression of Change (PGIC) was used to capture patients’ overall perception of treatment impact. Importantly, PGIC improved quite convincingly compared to placebo, suggesting that the perceived benefits of treatment outweighed any negative effects from adverse events. We believe this measure provides meaningful insight into the balance between efficacy and tolerability in this study. **We have emphasized this in the revised manuscript (line 296-299):**

***Cabergoline was well-tolerated, with side effects limited to mild and transient symptoms, including headache, dizziness, fatigue, and constipation. Notably, PGIC improved in the treatment group compared to placebo, indicating that patients perceived the benefits of treatment to outweigh any adverse effects experienced.***

6. Enhance the Title and Abstract to Increase Appeal
The title and abstract are crucial for capturing readers' attention. The current title is straightforward but lacks appeal. It is recommended to highlight terms such as “first evidence” or “significant efficacy” in the title. The abstract should also emphasize the study's innovative contribution compared to previous research.

We thank the reviewer for this encouraging recommendation. However, based on cautionary remarks raised by some of the other reviewers, we prefer to maintain the title ‘neutral’ but are willing to modify if the editor suggests so.

7. Refine Discussion of Limited Effects on Chronic Migraine
The discussion mentions that cabergoline has limited efficacy for chronic migraine, but the potential mechanism differences (e.g., varying responses to the CGRP pathway) or directions for treatment optimization are not deeply explored. Adding hypotheses or further discussion in this section would add depth. The authors could also enrich this section by citing related articles at appropriate positions in the discussion
(e.g., <https://journals.plos.org/plosone/article?id=10.1371/journal.pone.0304370>).

Thank you for raising these relevant points. The impetus for this investigator-initiated RCT was case-based evidence that cabergoline treatment of hyperprolactinemia prevented headache including migraine, which we also mention in the manuscript. Moreover, the dopamine receptor is implicated in migraine pathways and prolactin elevations are suggested to be associated with migraine both of which are also mentioned as hypotheses. In response to your previous point (3. Enhance the Rigor of Background Argumentation) we have added a paragraph providing further rationale for our study.

8. Improve Data Availability and Ethics Statements
The data sharing statement mentions that sensitive data cannot be fully disclosed but does not elaborate on how other researchers can access the data. It is recommended to provide a more specific data request process in the “Data Availability” section.

Our ethical committee only permits the sharing of anonymized data to ensure GDPR compliance. **We have added a “Data Availability” section to provide a more specific process for researchers to request access to anonymized data (line 376-377):**

***Data Availability: Anonymized data are available upon reasonable request.***

**Reviewer #5:** I thank the authors for testing, in an RCT, the effects of a molecule with significant metabolic effects, acting on the hypothalamic-pituitary axis.
In my opinion, the RCT yielded interesting results.

We thank the reviewer for their positive comments and for acknowledging the relevance and potential of our study.

I suggest that the authors evaluate the effect of cabergoline on glucose metabolism (1, 2) as a potential mechanism underlying the reduction in MMD in episodic migraine (3, 4).

**This is a very interesting point, and we have added a section in the discussion including appropriate citations (line 341-350):**

***Evidence from clinical studies suggests that impaired brain glucose metabolism and mitochondrial dysfunction, leading to a mismatch between the brain's energy supply and workload, contribute to migraine pathophysiology. Moreover, elevated inflammatory markers are frequently observed in individuals with migraine, indicating a state of chronic inflammation that may exacerbate migraine symptoms [7, 8]. This is of particular interest, as cabergoline treatment in patients with prolactinoma has been shown to significantly enhance insulin sensitivity and reduce inflammatory markers, independent of its effects on prolactin, LDL cholesterol, and BMI [9, 10]. Given the design of the current study, it is not possible to determine whether improvements in insulin sensitivity and reductions in inflammation contribute to the beneficial effects of cabergoline in migraine patients. These potential mechanisms, however, should be explored in future studies.***

If the authors had monitored weight, BMI, and HOMA-IR at baseline and after the intervention, it would be useful to include these data in the manuscript.

We thank the reviewer for highlighting the potential value of including weight, BMI, and HOMA-IR measurements to provide additional insights into the effects of cabergoline. Unfortunately, these measures were not collected in the current RCT; however, we plan to incorporate them in future studies.

The following point, in my opinion, requires correction:
You wrote: "Cabergoline significantly reduced MMD in episodic migraine and led to a significant global patient perceived improvement in both episodic and chronic migraine," but in the chapter Patient Global Impression of Change you stated: "PGIC significantly improved after cabergoline compared to placebo in the entire group, and in participants with episodic migraine. No significant difference was found in chronic migraine."
So, was there a significant improvement in PGIC in chronic migraine or not?
If my observation is correct, you should also revise the graphical abstract to reflect this aspect.

Thank you for pointing out this apparent ambiguity, and we apologize for the confusion. For clarification, the improvement in PGIC was significant in the **entire** group of cabergoline-treated patients compared to placebo. Post hoc analysis revealed that this positive effect was more pronounced in the subgroup of patients with episodic migraine, whereas no significant improvement was observed in the subgroup with chronic migraine. **We have revised the manuscript to state this more accurately (line 273-275):**

***While the primary outcome was not achieved, cabergoline significantly reduced MMD in episodic migraine and led to a significant global patient perceived improvement in the entire group of participants.***

References

1. Inancli SS, Usluogullari A, Ustu Y, Caner S, Tam AA, Ersoy R, et al. Effect of cabergoline on insulin sensitivity, inflammation, and carotid intima media thickness in patients with prolactinoma. Endocrine. 2013;44:193–9.

2. dos Santos Silva CM, Barbosa FR, Lima GA, Warszawski L, Fontes R, Domingues RC, et al. BMI and metabolic profile in patients with prolactinoma before and after treatment with dopamine agonists. Obes (Silver Spring). 2011;19:800–5.

3. Gross EC, Lisicki M, Fischer D, Sándor PS, Schoenen J. The metabolic face of migraine - from pathophysiology to treatment. Nat Rev Neurol. 2019 Nov;15(11):627-643.

4. Del Moro L, Rota E, Pirovano E, Rainero I. Migraine, Brain Glucose Metabolism and the "Neuroenergetic" Hypothesis: A Scoping Review. J Pain. 2022 Aug;23(8):1294-1317.

**Reviewer #6:** Thank you for the opportunity to review this manuscript. Overall this paper is clinically relevant and very well written. I have some minor points only.

Thank you for your positive feedback on the clinical relevance and quality of our manuscript.

Introduction: Please remove the last sentence on primary outcome (lines 81-82)

**We have removed the last sentence on the primary outcome (lines 81–82) as requested.**

Methods:
- Please write the year when the recruitement ended.

**Thank you. This has been added to the revised manuscript (line 91-92):**

***Recruitment started on the 5^th^ of September 2022 and ended on the 13^th^ of June 2023.***

- You excluded patients on dopamine antagonists, however some widely used antiemetics in migraine are dopamine antagonists, eg. metoclopramide and domperidone. Were these medications allowed? If yes, would you perform stratified analyses on patients who took antidopaminergic antiemetics vs those who didn't?

Thank you for highlighting the potential impact of dopamine antagonists, such as metoclopramide and domperidone, which are commonly used as antiemetics in migraine. These medications were not permitted in our study, as they could interfere with the dopamine-modulating effects of cabergoline. **We have clarified this exclusion criterion in the revised manuscript (line 102-104) to ensure this is clearly communicated:**

***The exclusion criteria encompassed pregnancy, breastfeeding, the use of dopamine antagonistic drugs (including antiemetics such as metoclopramide and domperidone), heart valve disease, severe untreated hypertension, and psychiatric disorders.***

- I couldn’t find any comment on power analysis. On which basis did you decide to include 36 participants only? Please explain. This is important given that, although non-significant, the carbegoline group had a higher reduction of MMD. Might it be that in a bigger population the difference would become significant? I guess that the biologic analgetic effect of placebo in pain studies (which you rightly touch on in the discussion section) makes reaching the statistical significance more difficult, which requires a careful planning.

This is a good point. The study was designed as a pilot trial, and the decision to include 36 participants (18 per arm) reflects its exploratory nature. The aim was to gather preliminary data on the efficacy and tolerability of cabergoline for migraine prevention, which could inform the design of a larger, adequately powered study. We agree that the placebo effect in pain and migraine studies presents a unique challenge, which may indeed increase variability, underscoring the importance of careful planning and sufficient sample sizes to detect statistically significant differences. A formal power calculation was not conducted prior to this trial due to the limited existing data on the efficacy of cabergoline for migraine prevention. However, based on the observed reduction in MMD in this study, we are now planning a larger RCT focusing on patients with episodic migraine. For the future trial, we are conservatively estimating a cabergoline effect size of 2.8 MMD and using the standard deviation of 4.1 days observed in this study, a sample size of 47 participants per arm would be required to achieve 90% power at a 5% significance level (α).

Discussion:
- When comparing absolute values of MMD reduction from your study to other studies, please be careful, as those results are likely based on populations with different baseline characteristics. I get the point and would not delete that part, but I would make the reader aware, that such comparisons should be interpreted with caution.

**We take the point and has modified the section accordingly (line 285-294):**

***When comparing the outcome of this study to contemporary migraine trials, the efficacy of cabergoline in episodic migraine in terms of a MMD reduction of 3.6 days*** ***appears clinically meaningful [11]. According to a recent review, the average reduction in MMD for anti-CGRP treatments versus placebo in episodic and chronic migraine were 1.9 and 2.2 days respectively, while reductions by other preventive treatments,*** ***including propranolol, metoprolol, onabotulinumtoxinA, topiramate, valproate, candesartan and amitriptyline, ranged from 0.9 to 1.7 days in episodic migraine and 1.8 to 2 days in chronic migraine [11]. It is important to interpret such comparisons with caution, as the populations in these studies likely differed in baseline characteristics; nevertheless, the cabergoline effect observed in episodic migraine is promising and warrants further investigation in a larger trial.***

- In the part where you discuss the risk of valvulopathy, please clarify whether there is a lack of evidence (eg due to scarcity of experience with the carbegoline), or whether there is evidence of a low risk of valvulopathy. As it reads now, I get the impression that there is no data available, which is different to evidence of a low risk. This is obviously important, because we do not want to gather experience on valvulopathy on migraine patients treated with carbegoline, given that the majority are young females.

This is a very important point. Indeed, there is ample experience from > 30 years of cabergoline treatment in hyperprolactinemia using the same low dose. No evidence of an increased risk of valvulopathy has been detected in these patients as recently reported and reviewed (*Stiles CE, Lloyd G, Bhattacharyya S, Steeds RP, Boomla K, Bestwick JP, et al. Incidence of Cabergoline-Associated Valvulopathy in Primary Care Patients With Prolactinoma Using Hard Cardiac Endpoints. J Clin Endocrinol Metab. 2021;106(2):e711-e20. doi: 10.1210/clinem/dgaa882. PubMed PMID: 33247916*). **We have tried to phrase this more precisely in the revised manuscript (line 303-311):**

***While long-term, high-dose cabergoline treatment (> 3 mg/day) in patients with Parkinson’s disease has been associated with an increased risk of fibrotic adverse events, including valvular heart disorder, the British Society of Echocardiography, the British Heart Valve Society, and the Society for Endocrinology, concluded that evidence suggesting low-dose dopamine receptor agonist treatment to be associated with abnormal valve morphology or dysfunction is extremely limited, and there is no evidence of clinically significant valve pathology, aside from isolated case reports with unresolved questions [12]. This is corroborated by recent studies including hard cardiac endpoints, which conclude that data do not support an association between low-dose cabergoline treatment and clinically significant valvulopathy [13] or other fibrotic adverse events [14].***

**Reviewer #7:** I have to congratulate the authors to perform such interesting IIT in a time when everyone is talking CGRP only.

The study is in gerenal sound and the manuscript is well written. Knowing that this is a pilot study, it would still be very interesting, if the authors performed a sample size calculation.

Thank you for your appreciation, which is very encouraging.

As this study was designed as a pilot or proof-of-concept study, a formal sample size calculation was not performed during the planning phase. However, based on the observed reduction in MMD in this study, we are now planning a larger RCT focusing on patients with episodic migraine. For the future trial, we are conservatively estimating a cabergoline effect size of 2.8 MMD and using the standard deviation of 4.1 days observed in this study, a sample size of 47 participants per arm would be required to achieve 90% power at a 5% significance level (α).

Still, as the study has failed the primary outcome, this is a negative study. This has to been stated clearly throughout the whole manuscript and especially in the abstract. After that some secondary endpoints and subgroup findings can be discussed and presented. But this has to be somewhat more conservative. All in all the numbers - especially for the subgroups - are rahter small to draw any conclusion. What is in the end confusing is the fact, that there was a difference in eM of 3.6 MMD and 2.3 in cM, but for the whole group only 1.3 (please explain, this is small number statistics!!)

We agree that the study did not meet its primary endpoint. This is clearly stated at the top of the discussion; however, we acknowledge that this was not stated in the abstract. **We have revised the abstract to meet the criticism (line 31-35):**

***No significant difference in monthly migraine days reduction was observed overall; in participants with episodic migraine (n= 20), the mean (SE) reduction in monthly migraine days from baseline to the last 28 days of the treatment period was -5.4 (1.3) (cabergoline) as compared to -1.8 (0.9) (placebo) (p=0.04) [odds ratio: 0.79 (95% CI 0.65 – 0.95), p=0.014].***

We also agree that the small numbers, particularly in the subgroups, limit the ability to draw definitive conclusions. **This limitation has been further emphasized in the discussion, and we propose a larger study as a necessary next step to confirm these findings (line 351-359):**

***The major strength of our study lies in its randomized, placebo-controlled design, which minimizes bias and enhances validity. However, the small sample size remains a limitation, increasing the risk of random variability and the potential of overestimation or underestimation of effects. Subgroup analyses in smaller populations are particularly susceptible to spurious findings, underscoring the importance of cautious interpretation of these results. ... A larger study is needed to substantiate our findings.***

Regarding the change in the number of MMD from baseline, in patients with episodic migraine mean (95% CI) difference in the cabergoline group compared to placebo was -3.6 (-7.0, -0.2) (p = 0.04), whereas in patients with chronic migraine, the median (IQR) difference in the cabergoline group compared to placebo was -1.5 (-8.3, 3) (NS) and not 2.3 as stated by the reviewer.

Minor:
- line 49: what is the reference here. The life-time prevalance of migraine is much higher. 15% corresponds rather to point-prevalence.

Thank you for your comment regarding the lifetime prevalence of migraine. You are correct that the 15% prevalence cited corresponds more closely to point prevalence rather than lifetime prevalence. According to the data from Stovner et al., 2022, (PMID: 35410119), the global lifetime prevalence of migraine is estimated at 17.5% (95% CI: 14.6–21.0%) for both genders, with a higher prevalence among females (21.0%) compared to males (11.6%). **We have updated the manuscript accordingly and included the Stovner et al., 2022, as the reference (line 48-50):**

***The estimated global lifetime prevalence is approximately 17.5%, with a higher frequency in women (21.0%) compared to men (11.6%).***

- line 168: This should maybe read "Fifty-four", otherwise the sum is not correct

Thank you for the thorough proofreading. **We have corrected this.**

- line 271: this is not fully true, in some studies also RWE effects in cM were much higher

We acknowledge that some RWE studies have demonstrated higher treatment effects in chronic migraine populations compared to what is often observed in controlled clinical trials. Considering this, **we have pointed out in the revised discussion that, while patients with chronic migraine commonly exhibit a lower treatment response, this is not always the case (line 278-282):**

***Our study population comprised only 13 participants with chronic migraine, which is noteworthy since this group commonly exhibits a lower treatment response [15] also to CGRP-targeting therapies [16], but treatment responses in chronic migraine may vary, underscoring the need for further research in this subgroup.***

- line 276: if you divert eM and cM in the others you also should do this for your data: 3.6 and 2.3 days (than there is less difference in cM)

We have chosen to focus on the significant reduction in MMD observed in participants with episodic migraine (3.6 days), as no significant reduction was observed in participants with chronic migraine. The lack of statistical significance in the chronic migraine subgroup may be attributed to the small sample size or a true lack of efficacy in this subgroup. We acknowledge, however, that the limited sample size for chronic migraine participants restricts our ability to draw definitive conclusions.

Please explain/discuss:
- Line 233: why was there no change in MIDAS/HIT-6

We have no clear answer for this good question. When we survey the literature, significant symptomatic improvements based on MIDAS are rare in placebo-controlled clinical migraine trials with much larger sample sizes (e.g: Journal of Neurology (2024) 271:5762–5777). We prefer not to speculate further on this in our revised manuscript when considering the small sample size.

- Line 236: why did so many guess correctly?
- It seems that Cabergoline can not be blinded? Differences in AEs, so many guess correctly, difference in PGIC? !

Thank you for raising these relevant questions. In general, the incidence of AE was low both after cabergoline and placebo, so we do not believe that AE was driving the ability to ‘guess correctly’ in the cabergoline group. The prominent improvement in PGIC in the cabergoline group (mainly driven by the subgroup with episodic migraine) suggest that the higher prevalence of guessing correctly after cabergoline relates to (impression of) a beneficial effect. If the latter is correct, it emphasizes the challenge of complete blinding when the outcome is pain (headache). The perfect preventive migraine drug would permanently eliminate MMD to zero, which would likely translate into a nearly 100% of correct guessing in the active group as compared to (the usual) 20% in the placebo group. This must be an inherent challenge and we prefer not to elaborate more on this in the revised manuscript.

- Please discuss possible issues of MOH in the long-term when acting and 5HT-receptors!

Point taken. Based on our case-based experience with hyperprolactinemia patients (and concomitant headache including migraine) treated with cabergoline over many years/decades, we see no obvious sign of MOH. However, the risk merits mentioning in the manuscript. **We have revised accordingly (line 299-302):**

***Although the major mechanism of action for cabergoline is via the D2 receptor, cabergoline is also a 5-HT receptor ligand, which could introduce a risk of MOH similar to what is reported after long-term frequent use of triptanes [17]. This merits consideration in any future trial.***

**Reviewer #8:** Dear Author,

I think this is a very interesting study that suggests another possibility for migraine prophylaxis, so it is worth publishing. However, I would like to point out several points where more information should be added.

Thank you for your positive feedback. It is very encouraging.

Firstly, when did recruitment end? The 13th of June 2023? If so, please add "2023" to the text (line 87).

You are correct that recruitment ended on the 13th of June 2023. **We have revised accordingly.**

The second is about the recorded "migraine" attacks. A migraine day seems to be defined by an electronic diary recorded by the participants themselves. How did the authors confirm that these pain attacks were really migraine attacks? Especially in CM patients, attacks can have several different patterns. Were there no participants with multiple types of headaches, such as tension-type headache?

Accurate classification of migraine days and the potential for multiple headache types in this study population are important considerations. In the electronic headache diary, participants recorded detailed headache characteristics, including descriptions of pain intensity, duration, associated symptoms (e.g., nausea, photophobia, phonophobia), and the use of acute medications. These criteria align with the International Classification of Headache Disorders (ICHD-3) diagnostic framework to help distinguish migraine attacks from other headache types, including tension-type headaches. We acknowledge, however, that distinguishing between headache types, particularly in patients with chronic migraine, can be challenging due to the overlap in symptom patterns. While we relied on participant-reported data to classify migraine days, we recognize that this approach may not fully account for potential diagnostic ambiguities or mixed headache presentations.

Also, in line 168-169, "Forty-five patients" should be "Fifty-four patients".

Thank you for the thorough proofreading. **We have corrected this.**

The last point concerns the discussion. Although I agree with the significant efficacy of cabergoline shown in this study, it was still unclear whether this can be generalized beyond the sex difference, as most participants were female. In addition, the authors refer to an interesting change that occurred in female mice, reported in one of the references. Therefore, I think that the suggested efficacy of the present study should be limited to female patients. I will not deny possibility of efficacy in male patients; however, it is not able to refer that in this study. This is one of the limitations of this study.

Thank you for pointing out the potential difference in efficacy between the sexes. We acknowledge that the efficacy of cabergoline demonstrated in this study may primarily apply to female patients, given the sample composition. While it remains possible that cabergoline could also be effective in male patients, this cannot be concluded from the current study. We agree that the predominance of female participants in this study limits the generalizability of the results with respect to male patients. **This is an important limitation that we have now addressed more explicitly in the discussion section (line 357-359):**

***Finally, the predominance of female participants limits the generalizability of the results, and our findings should therefore be interpreted with caution when applied to male patients.***

**Reviewer #9:** The sample size is too small, the results are not convincing, and the reduction in MMD days alone is not convincing. More pain characteristic needs to be provided, such as pian duration, changes in VAS scores, and changes in accompanying symptoms.

We thank the reviewer for taking time to review our manuscript.

We acknowledge that the sample size is a limitation of this study, as noted in the manuscript. However, this study was designed as a pilot trial to explore the potential efficacy and safety of cabergoline in migraine management, laying the groundwork for larger, more definitive trials. **We have revised the discussion to emphasize this (line 351-359):**

***The major strength of our study lies in its the randomized, placebo-controlled design, which minimizes bias and enhances validity. However, the small sample size remains a limitation, increasing the risk of random variability and the potential of overestimation or underestimation of effects. Subgroup analyses in smaller populations are particularly susceptible to spurious findings, underscoring the importance of cautious interpretation of these results. … A larger study is needed to substantiate our findings.***

While the reduction in MMD is a key endpoint, we recognize the value of additional pain characteristics in providing a more comprehensive understanding of treatment effects. On the other hand MMD is close to a gold standard and the reduction in MMD in patients with episodic migraine after cabergoline treatment was very substantial and merits to be tested in a larger trial. Finally, we did record very robust and positive effects of cabergoline on PGIC in the entire group, which we also consider encouraging. The concept of conducting a pilot trial is acknowledged in the guidelines from the International Headache Society (Headache Classification Committee of the International Headache Society (IHS) The International Classification of Headache Disorders, 3rd edition. Cephalalgia. 2018;38(1):1-211. doi: 10.1177/0333102417738202. PubMed PMID: 29368949) and we argue that our pilot data merit to be published and subsequently tested in a larger RCT.
