# Supplementary material for: Cabergoline as a preventive migraine treatment: A randomized clinical pilot trial
Source: PLoS One. 2025 Apr 1;20(4):e0320937. doi: 10.1371/journal.pone.0320937 (PMC11960899; doi:10.1371/journal.pone.0320937)
Supplement: Attachment — Submitted filename: comments.docx [file pone.0320937.s003.docx]

Thank you for inviting me to review this manuscript. I believe the authors have conducted a clinical study that is beneficial for migraine patients. Its quality is sufficient to be published after revisions. Below are my suggestions:

1. Further Discussion on Study Limitations

Although the discussion section mentions the small sample size as a limitation, other potential influencing factors (e.g., short follow-up duration, sample selection bias) should be explored further. Suggestions for addressing these limitations, such as conducting multicenter or larger-scale studies in the future, can also be included.

2. Clarify Titles and Annotations in Figures

The data presented in the figures are central to the manuscript, particularly Figures 2 and 3. Strengthening the descriptions in the figure titles and annotations can facilitate readers' quick understanding while highlighting the key conclusions of the study.

3. Enhance the Rigor of Background Argumentation

The background section mentions that "the relationship between dopamine and migraines remains controversial," but the cited references are not sufficiently comprehensive. Recent findings on the role of dopamine in migraine mechanisms should be incorporated to improve the relevance and credibility of the references.

4. Improve Readability of Statistical Method Descriptions

The statistical analysis mentions the use of linear regression models and non-parametric tests, but the rationale for selecting these methods is not well explained. Additional clarification on why these methods were chosen and how potential confounding factors were controlled would enhance the methodological clarity.

5. Supplement Detailed Analysis of Adverse Events

While the types and frequencies of adverse events are listed, their impact on patients has not been detailed. Adding analyses based on scales or patient feedback to quantify the impact of adverse events on quality of life could strengthen this aspect of the manuscript.

6. Enhance the Title and Abstract to Increase Appeal

The title and abstract are crucial for capturing readers' attention. The current title is straightforward but lacks appeal. It is recommended to highlight terms such as “first evidence” or “significant efficacy” in the title. The abstract should also emphasize the study's innovative contribution compared to previous research.

7. Refine Discussion of Limited Effects on Chronic Migraine

The discussion mentions that cabergoline has limited efficacy for chronic migraine, but the potential mechanism differences (e.g., varying responses to the CGRP pathway) or directions for treatment optimization are not deeply explored. Adding hypotheses or further discussion in this section would add depth. The authors could also enrich this section by citing related articles at appropriate positions in the discussion

(e.g., https://journals.plos.org/plosone/article?id=10.1371/journal.pone.0304370).

8. Improve Data Availability and Ethics Statements

The data sharing statement mentions that sensitive data cannot be fully disclosed but does not elaborate on how other researchers can access the data. It is recommended to provide a more specific data request process in the “Data Availability” section.
